# Ergonomics and Work-Related Musculoskeletal Disorders: Characteristics Among Female Interventionists

**DOI:** 10.7759/cureus.18226

**Published:** 2021-09-23

**Authors:** Emily Barnard, Kristin Sheaffer, Sarah Hampton, Megan L Measel, Ahmed Farag, Cathyrn Shaw

**Affiliations:** 1 Medicine, Mercer University School of Medicine, Savannah, USA; 2 Biomedical Engineering, Georgia Institute of Technology, Atlanta, USA; 3 Interventional Radiology, Baylor University Medical Center, Dallas, USA

**Keywords:** ergonomic training, ergonomics, gender differences in ergonomics, female proceduralists, women in surgery, interventionist, interventional radiology, work-related injury, work-related musculoskeletal disorders

## Abstract

Interventional radiology is a procedural specialty that performs minimally invasive operations under image guidance. Currently, there are inadequate ergonomic protocols for work-related musculoskeletal disorder (WMSD) prevention in interventional radiology (IR), and there is a paucity of information discerning gender differences in WMSDs. This article reviews current literature that addresses WMSDs in female physicians practicing interventional and fluoroscopic procedures, including interventional radiology, interventional cardiology, electrophysiology, vascular surgery, orthopedic surgery, neurosurgery, and gastroenterology. We searched PubMed and EBSCOhost databases for ergonomic studies that reported female physician WMSDs in the specialties listed above. After a thorough evaluation for inclusion based on eligibility criteria, 11 studies were included. From this search, there was poor female representation, averaging 25.7% of respondents. Several characteristics identified across the studies were that women were generally shorter, wore smaller glove sizes, and were younger than their male colleagues. Seventy-two percent of female proceduralists reported WMSDs versus 46.6% of their male colleagues. Additionally, women may experience more upper extremity pain than lumbar pain, which men commonly reported. Potential contributing factors to WMSDs are the size and design of procedural tools and the possible predisposition of female physicians to experience upper extremity WMSDs while performing the same operations as men. As more women enter medicine and pursue careers in procedural fields like interventional radiology, it is essential to address these discrepancies and develop ergonomically sound solutions for women.

## Introduction and background

Interventional radiology (IR) is a procedural specialty that performs minimally invasive operations under image guidance. The occupational risk factors frequently encountered by interventional radiologists are radiation exposure and work-related musculoskeletal disorders (WMSDs) [1-3]. While precautionary conventions to reduce radiation exposure are well-established for interventional procedures, there are inadequate ergonomic protocols for WMSD prevention in IR [1-2,4-7]. Due to the limited number of studies addressing work-related injury in IR, data concerning ergonomics are often extrapolated from similar specialties that perform interventional and fluoroscopic procedures. Interventional cardiology, electrophysiology, vascular surgery, gastroenterology, orthopedic surgery, and neurosurgery are specialties that perform interventional procedures which frequently utilize fluoroscopy. Among these fields, physicians commonly reported WMSDs of the cervical spine and lumbar spine, which seemingly correlated with monitor viewing, wearing lead protection, poor posture, and instrument design [8-17]. The considerable increase in cervical pain among interventional cardiologists was attributed to the use of fluoroscopic monitors (*P* < 0.002) [9]. Likewise, when comparing electrophysiologists to noninterventional cardiologists, there was a noticeably greater prevalence of cervical spondylosis (20.7% vs 5.5%, *P* = 0.033) and lumbar spondylosis (25.9% vs 16.7%, *P* = 0.298) [10]. Forty-eight to sixty-seven percent of endoscopists reported musculoskeletal injury, with 32-74% contributing the pain to endoscopic retrograde cholangiopancreatography (ERCP) [14,15]. The most common areas of pain were the neck (24-46%), lower back (17-57%), and hand (33%) [14,15]. The most common injury was de Quervain’s tenosynovitis (16%), followed by cervical radiculopathy (12%) [14]. Among orthopedic surgeons, repetitive flexion with torquing during procedures was the highest predictor for developing WMSDs after adjusting for age, BMI, and exercise habits (*P* = 0.008) [8]. Furthermore, wearing lead aprons was associated with more frequent back pain, where 74.2% of orthopedic surgeons reported the aprons as less-than well-fitting, and 83.9% stated the lead was too large [16].

The number of women entering the medical field and pursuing procedural specialties is continually increasing. In 2019, the Association of American Medical Colleges (AAMC) reported that for the first time, there were more women (50.5%) enrolled as US medical students than men (49.5%) [18]. There has been a progressive growth in female medical students from 46.5% in 2015 to 50.5% in 2019 and an increase in the number of applicants and matriculants by 1.1% from 2018 to 2019 [18]. Female interventional radiologists represented 8.2% of all interventional radiologists nationally in 2019 [19]. Overall, there is a lack of ergonomic data in IR, and there is a paucity of information discerning gender differences in WMSDs [1]. Considering the limited female physician responses among most ergonomic studies available, we reviewed the current literature that specifically considers WMSDs in women practicing in interventional and fluoroscopic fields. While women in IR are under-represented, there is a steady increase in women entering medicine and pursuing careers in IR. Ergonomic differences must be identified so that adjustments can be made now, as there will be more female proceduralists in the future.

## Review

Methods

Search Strategy 

We performed a systematic review of the literature following the Preferred Reporting Items for Systematic Reviews and Meta-Analyses (PRISMA) guidelines to identify journal articles that addressed ergonomics and WMSDs among females working in IR and comparable specialties [20]. We conducted an electronic search through EBSCOhost (https://www.ebsco.com/products/research-databases) and PubMed (https://pubmed.ncbi.nlm.nih.gov). Search terms included (Ergonomics [title/abstract]) AND Interventional Radiology, Interventional Cardiology, Electrophysiology, Vascular Surgery, Neurosurgery, Orthopedic Surgery, Fluoroscopy, and Endoscopy; (Musculoskeletal injuries OR injury OR pain OR symptoms [title/abstract]) AND Interventional Radiology, Interventional Cardiology, Electrophysiology, Vascular Surgery, Neurosurgery, Orthopedic Surgery, Fluoroscopy, and Endoscopy. The search boundaries were set to 2000-2021. Abstracts found within the search terms were screened using the selection criteria by two authors (EB, KS) for their appropriateness for inclusion in this study. Any incongruities regarding article inclusion were presented to the remaining authors for a final consensus.

Eligibility Criteria 

Abstracts of all the studies were inspected to confirm that appropriate information was present for review. The articles were considered eligible if they included self-reported ergonomic assessments or questionnaires, physicians in the specialties listed in our search, and contained responses from female physicians. We excluded studies that were not published in a peer-reviewed journal or articles with unobtainable text.

Data Extraction and Analysis 

All the authors independently analyzed the studies found through PubMed and EBSCOhost. The data were acquired from the text, graphs, tables, and figures present in the articles and collected with Microsoft Excel 2020 version 16.45 (Microsoft Corp, Redmond, Washington). Quantitative means were calculated for several data points among the studies included.

Results 

Study Selection

**Figure 1:**
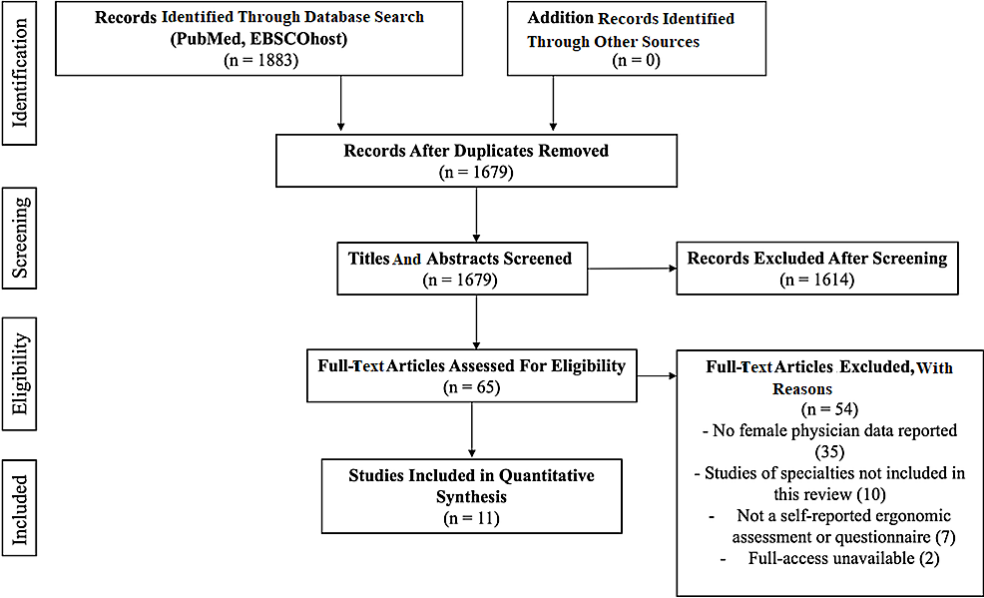
PRISMA Flow Diagram PRISMA: Preferred Reporting Items for Systematic Reviews and Meta-Analyses

The database search through PubMed and EBSCOhost generated 1883 results. After removing 204 duplicate studies, 1679 remained to be screened for eligibility. After the additional screening of titles and abstracts, a total of 65 articles on ergonomics and work-related musculoskeletal injuries remained. The 65 studies were further reviewed through a full-text screening to evaluate if they met the eligibility criteria. In the full-text screening, articles were searched for the words: female, woman, women, sex, and gender to identify if gender demographics were collected from the respondents. After applying exclusion criteria, eleven studies remained which met eligibility criteria established by the authors [2,3,21-29]. These articles contained reports of female physician WMSDs in interventional and fluoroscopic specialties. The PRISMA flow diagram illustrating the screening and exclusion process is displayed in Figure 1.

Female Physician Representation and Characteristics 

The average percentage of female physician respondents from these studies was 25.7% (Table 1) [2,3,21-29]. In one study, Orme et al. distributed a survey among the Mayo Clinic IR and interventional cardiology (IC) facilities to address radiation exposure and WMSDs among their medical staff, including nurses, technicians, residents, fellows, and attendings [3]. This study received a female response rate of 67%. However, the actual number of female physicians and trainees was not provided. The low rate of female responses among these studies is likely due to the lower female representation in these specialties. Two studies noted that female respondents generally wore smaller gloves, were shorter, and were younger than their male colleagues (Table 2) [21,22].

**Table 1:** Ergonomic Studies Including Female Physicians *Orme et al. include responses from female ancillary staff (registered nurses, technician/technologists) [3]. CR: category ratio; MSD: musculoskeletal disorder; WMSD: work-related musculoskeletal disorder; MSK: musculoskeletal

Study	Specialty	Methods (Survey; Responses)	Percentage of Female Responses (Female/Total Respondents)	Findings of Female Respondents
Morrison et al. [2]	Interventional radiology	(Electronic survey including the Nordic Musculoskeletal Questionnaire; 640)	11% (69/640)	Multivariate analysis identified the female gender as a factor associated with a higher risk of moderate to severe MSDs.
Pawa et al. [21]	Gastroenterology, endoscopy	(38-item electronic survey on demographics, workload, and musculoskeletal injury; 1698)	34.3% (583/1698)	Women were noted to wear a smaller glove size, be shorter and younger than their male colleagues. Women reported a more significant number of work-related MSDs. Female physicians noted new-onset or worsening of MSDs while pregnant.
Fram et al. [22]	Orthopedic surgery	(21-item online survey on demographics, physical symptoms and treatment, perceptions, and instrument-specific concerns; 204)	58.6% (119/204)	There was a significant difference between male and female height and glove size. Female surgeons reported more symptoms, obtained treatment for WMSDs, and reported increased difficulty using orthopedic instruments.
Morais et al. [23]	Gastroenterology, endoscopy	(39-item electronic survey on demographics, endoscopy-related musculoskeletal injury, and workload; 171)	55% (94/171)	The female gender was a risk factor for musculoskeletal injury and severe pain.
Byun et al. [24]	Gastroenterology, endoscopy	(Self-reported questionnaire on demographics, duration of practice, musculoskeletal disorders, postures, and habits during endoscopy; 55)	32.7% (18/55)	There was a higher rate of MSDs among female endoscopists.
Alzahrani et al. [25]	Orthopedic surgery	(Electronic survey on demographics and work-related injury; 402)	24% (97/402)	Female surgeons had a higher risk of exacerbation of a previous MSK injury.
Davila et al. [26]	Vascular surgery	(35-item electronic survey quantifying pain before, during, and after surgical procedures using the Borg scale. There were additional questions on workload, burnout, and professional satisfaction; 224)	17% (38/224)	There was no difference between male and female genders in physical discomfort before, during, or after procedures.
Mavrovounis et al. [27]	Neurosurgery	(Electronic 38-item survey on demographics, WMSDs, procedures performed, and views on ergonomics; 409)	17.6% (72/409)	There was no association with the development of work-related MSDs between gender.
Gadjradj et al. [28]	Neurosurgery	(Electronic 29-item questionnaire on demographics, workload, procedures performed, and WMSDs; 417)	16.1% (67/417)	There was no significant difference between male and female genders for physical discomfort.
Wohlauer et al. [29]	Vascular surgery	(Electronic survey including Maslach Burnout Inventory, Borg CR10 scale, demographics, and wellness questions; 736)	16.4% (121/736)	There was no difference in the development of WMSDs between male and female genders for open, endovascular, or endovenous procedures.
Orme et al. [3]	Interventional radiology and interventional cardiology	(Electronic survey on demographics, work-related musculoskeletal pain, radiation exposure; 1543)	67%^* ^(1034/1543)	Women were more likely to report WMSDs. The highest rates of WMSDs were among technicians.
Average Percentage of Female Physician Respondents			25.7% (1278/4956)	

**Table 2:** Female Physician Characteristics *The numerical glove sizes were not listed in Pawa et al. [21]. WMSD: work-related musculoskeletal disorder

Characteristics	Study	Assessment		P-Value
Odds Ratio (OR) for Increased Risk of WMSDs in Female Physicians	Morrison et al. [2]	3.35		< 0.001
	Morais et al. [23]	2.443		0.018
Gender		Female	Male	
Average Height (inches)	Pawa et al. [21]	64.6 in	70.1 in	< 0.001
	Fram et al. [22]	65.3 in	70.8 in	< 0.001
Average Age (years)	Pawa et al. [21]	45.4 years	55.3 years	< 0.001
Average Glove Size	Pawa et al. [21]	Extra-small to medium (96.7%)^*^	Large to extra-large (73.0%)^*^	< 0.001
	Fram et al. [22]	Size 6.5	Size 8	< 0.001

Reports of WMSDs 

Self-reported physical discomfort among all respondents averaged at 72.4%, and the locations of WMSDs were most often reported in the lumbar spine, cervical spine, shoulder, and hands (Table 3) [2,3,21-29]. In Table 4, mean percentages of the areas described in this review were lumbar spine (47.2%), cervical spine (44.6%), shoulder (42%), arm and elbow (14.7%), wrist (34.2%), thumb (30%), and hand and fingers (55.3%) [2,21,23-29]. In six studies, the rate of female-reported pain was 72% compared to 46.6% for males (Table 3) [3,22-25,28]. From this data, there was no difference between the total self-reported WMSDs (72.4%) and the female reports of work-related injury (72%). However, there is some variability between the female (72%) and male-reported (46.6%) rates of WMSDs [3,22-25,28].

**Table 3:** Self-Reported WMSDs *Percentage includes female ancillary staff. **Reports of severe musculoskeletal pain. ***Pain reported after procedures. NR: not reported; WMSD: work-related musculoskeletal disorder

Study	Percentage of All Respondents (Self-Reported WMSDs/ Total Respondents)	Percentage of Female Respondents (Female Self-Reported WMSDs/Total Female Respondents)	Percentage of Male Respondents (Male Self-Reported WMSDs/Total Male Respondents)
Morrison et al. [2]	88% (563/640)	NR	NR
Orme et al. [3]	54.7% (844/1543)	71%*(734/1034)	56% (285/509)
Pawa et al. [21]	75.2% (1277/1698)	NR	NR
Fram et al. [22]	69.8% (141/204)	86.5% (103/119)	45.2% (38/84)
Morais et al. [23]	69.6% (119/171)	59.6% (56/94)	40.3% (31/77)
Byun et al. [24]	89.1% (49/55)	61.1%^** ^(11/18)	40.5%^** ^(15/37)
Alzahrani et al. [25]	67% (269/402)	71% (69/97)	66% (201/305)
Davila et al. [26]	96.8%^*** ^(217/224)	NR	NR
Mavrovounis et al. [27]	88% (360/409)	NR	NR
Gadjradj et al. [28]	73.6% (307/417)	82% (55/67)	17.7% (62/350)
Wohlauer et al. [29]	76% (559/736)	NR	NR
Average Reports of WMSDs	72.4% (4705/6499)	72% (1028/1429)	46.4% (632/1363)

**Table 4:** Location of WMSDs Among All Respondents *Percentages were summed for the locations of the left and right shoulders and wrists. **Experiencing pain during procedures. ***Percentages summed from endovascular and endovenous procedures. NR: not reported; WMSD: work-related musculoskeletal disorder

Study	Lumbar	Cervical	Shoulder	Arm/Elbow	Wrist	Thumb	Hand/Fingers
Morrison et al. [2]	61% (390/640)	56% (358/640)	46% (294/640)	NR	NR	NR	NR
Pawa et al. [21]	52.6% (893/1698)	59% (1002/1698)	47% (798/1698)	NR	45% (764/1698)	63.3% (1075/1698)	56.6% (961/1698)
Morais et al. [23]	NR	30.4% (52/171)	NR	NR	NR	29.2% (50/171)	NR
Byun et al. [24]	NR	NR	21.8%^* ^(12/55)	NR	23.6%^* ^(13/55)	NR	16.4% (9/55)
Alzahrani et al. [25]	28.60% (115/402)	10% (40/402)	13% (52/402)	15.4% (62/402)	10% (40/402)	NR	NR
Davila et al. [26]	14.5% (32/224)	12.2% (27/224)	NR	NR	NR	NR	NR
Mavrovounis et al. [27]	47.4% (194/409)	48.7% (199/409)	34% (139/409)	14.2% (58/409)	14.7% (60/409)	NR	NR
Gadjradj et al. [28]	33.8%** (141/417)	41.5%** (173/417)	24.9% ** (104/417)	NR	NR	NR	NR
Wohlauer et al. [29]	65%*** (478/736)	36.8%^*** ^(271/736)	8.7%^*** ^(640/736)	NR	NR	NR	NR
Average Percentage	47.2% (2243/4752)	44.6% (2122/4652)	42% (2039/4357)	14.7% (120/811)	34.2% (877/2564)	30% (1125/3738)	55.3% (970/1753)

In Alzahrani et al., female pediatric orthopedic surgeons had a higher rate of requiring time off due to WMSDs (36% vs. 29%) [25]. Additionally, exacerbation of a previous WMSD was more common among female surgeons (*P* < 0.05) [25]. Women employed in interventional facilities were also more likely to note a history of WMSDs (71%; *P* < 0.001) [3]. Byun et al. reported a higher response of severe musculoskeletal pain amongst female endoscopists when compared to males (61.1% vs. 40.5% *P* = 0.152) [24]. According to the two studies, the female gender was associated with a higher risk of severe WMSDs (OR 3.35; *P* < 0.001 [2] and OR 2.443; *P* = 0.018) (Table 2) [23]. In contrast, five studies in the specialties of vascular surgery, neurosurgery, and gastroenterology reported no statistical difference in the likelihood or the prevalence of WMSDs between genders [21,26-29]. However, of these five studies, Pawa et al. did comment that women reported a statistically higher mean number of endoscopic-related injuries (ERI) compared to men (5.9 vs. 5.3; *P* < 0.001) [21].

From this data, it is observed that women experience occupational pain at a similar rate to their male coworkers [3,22-25,28]. It is uncertain whether women have a greater risk for developing WMSDs when compared to men. Of note, the studies that reported no statistical difference in the development of WMSDs did not analyze the locations of pain experienced by female physicians or explore other characteristics regarding the female respondents [21,26-29].

Female Physician WMSDs and Ergonomic Considerations

Women tend to have smaller hands and wear smaller gloves than men in surgical fields [21,22]. Fram et al. found that female orthopedic surgeons were more likely to report work-related musculoskeletal symptoms than their male colleagues, which was attributed to the use of surgical instruments (87.3% vs. 45.2% *P* < 0.001) [22]. Furthermore, female surgeons significantly reported negative attitudes regarding orthopedic instruments and reported increased difficulty using these tools (*P* < 0.001 for both measures) [22]. The current size and design of available procedural tools should be considered when assessing workflow efficiency and the prevalence of WMSDs. 

The location of WMSDs varies with gender, where women may experience upper extremity pain in the shoulders and wrists, while men often experience lumbar spine and cervical spine pain [1,21,22]. Due to instrument size, women had a more significant concern for developing WMSDs, specifically hand injury [22]. In Pawa et al., men were more likely to report ERI due to lead aprons causing back pain, whereas women reported increased frequency of injury from duodenoscope elevator use, a hand-held device (*P* < 0.001 for both measures) [21].

Table height is essential when addressing ergonomics in interventional procedures and fluoroscopy [1]. Optimal ergonomic table height for interventionists is at the level of the physician’s elbows [1]. Shorter physicians tend to compensate for their height by using step stools when the table cannot be adjusted to an optimal ergonomic height [1]. Step stool use is considered a dangerous and unsatisfactory ergonomic compromise, often overcompensating the height deficit, increasing the risk of losing balance or falling. Additionally, having nonadjustable beds or monitors can increase the amount of torquing and cause poor posture, further exacerbating the risk for WMSDs, specifically in the back, neck, and shoulders [1,21]. For women, nonadjustable equipment was a significant contributor to ERI, and improper positioning during endoscopic procedures was related to the development of severe musculoskeletal injury [21,24]. Though women reportedly experience less back and neck work-related injury, improper monitor viewing, and overcompensation with step stools can add additional strain, contributing to the more often experienced upper extremity pain [1,21].

A unique consideration for women is the effects of pregnancy on the development of WMSDs. Pawa et al. reported data regarding pregnant endoscopists and their experiences with ERI [21]. Out of the female responses, 19.6% reported a history of being pregnant while in practice. During their pregnancies, 78.9% reported a new-onset injury, and 70.2% reported worsening pre-existing injuries. Several biomechanical adaptations occur during pregnancy that could increase the risk of WMSDs, which may contribute to this finding [30].

Discussion

WMSDs Among Female Physicians

Potential factors that may contribute to the WMSDs experienced by female physicians are height and glove size [21,22,31,32]. Wearing a smaller glove size (≤ 6.5) was associated with increased difficulty in using laparoscopic instruments, scissors, and staplers compared to a larger size (≥ 7.0) [22,31,32]. Consequently, based on the distribution of glove sizes among the sample population of surgeons in Berguer and Hreljac, 87% of females and 22% of males may encounter difficulty with instrument use and are at increased risk of a hand injury [31]. Female surgeons in Sutton et al. reported that laparoscopic staplers were too large for proper grasp and utility (78 vs. 28%, OR 8.85; *P* < 0.001), and that 84% of women and 73% of men in the study stated that instrument design was a potential cause of physical discomfort in the operating room [32]. This correlates with the reports of pain using orthopedic tools and increased physical symptoms reported by women in Fram et al. [22]. Interestingly, Sutton et al. noted that women experienced significantly more shoulder pain than men wearing the same glove size (77 vs. 27%; *P* = 0.004) [32].

In a study conducted by Armijo et al., data on surface electromyography of the surgeon’s dominant upper limb was utilized to identify the quantity of muscle activation during laparoscopic procedures [33]. Of the 18 surgeries recorded, eight of the surgeons were female. There was a statistical increase in the muscle activation of the shoulder and wrist in the female physicians compared to men performing the same movements. There was a significant increase in maximal voluntary contraction of the upper trapezius, flexor carpi radialis, and extensor digitorum. Postoperatively, there was a higher self-reported sensory and cognitive fatigue score in women after the surgeries, while men reported no changes in fatigue [33].

Further reviews analyzing WMSDs in manufacturing workers have found that women are more likely to develop upper extremity pain and fatigue while performing the same industrial work as men [34,35]. Nordander et al. showed that female workers in rubber manufacturing and mechanical assembly plants had higher maximal voluntary contractions of the trapezius and forearm extensors when compared to men [34]. Moreover, there was a higher prevalence of neck and upper extremity WMSDs among female workers when similar working postures and movements were performed [34]. These findings correspond with the reports by Armijo et al. described previously [33]. Slopecki et al. found that women had a significantly higher percentage change in the anterior deltoid when measuring muscular activity with surface electrodes when the anthropometric load was included as a covariate [35]. These results suggested that women often experience work-related upper extremity pain than the lumbar spine and cervical spine pain reported by men when performing the same work. The application of anthropometry should be considered when analyzing ergonomic differences and developing medical device design.

Though IR procedures utilize catheter and wire-based tools versus laparoscopic instruments, there is still a concern for developing upper extremity pain in female interventionists. When assessing the ergonomic problems in hepatic arterial catheterization and gastrointestinal stent placement, two standard IR procedures, Shinohara identified that the usage of small caliber catheters was associated with increased neuromuscular fatigue [36]. Catheter manipulation requires twisting the body, neck, arm, and wrist, increasing the risk of WMSDs of the upper extremity [36]. Furthermore, in a study assessing the utility of a FlexArm (Philips Medical Systems Nederland B.V., Best, The Netherlands), there was a noted reduction in physical discomfort, the need for table repositioning, and the risk of displacing access sheaths [37]. Compared to a traditional ceiling-mounted C-arm, the flexible C-arm system demonstrated marked improvement in physical comfort, which was most significant in the arms, hands, shoulders, and upper back (*P* < 0.001 for all data points) [37]. Factors that might contribute to upper extremity WMSDs for female interventional radiologists are table height, monitor positioning, and posture [2,21,24]. Sutton et al. reported discomfort due to the table height in 43.75% of the women [21,32]. The use of nonadjustable equipment can significantly contribute to ERI, and there are risks to using step stools, such as work-related injury from overcompensation [1,21,24]. Nonadjustable tables and monitors increase torquing and poor posturing, further exacerbating the risk for WMSDs, specifically the back, neck, and shoulder [1,21].

Regarding the increase in WMSDs in pregnant physicians, several biomechanical factors may contribute to this finding [21,30]. During pregnancy, there are natural physiologic adaptations to the female anatomy, such as increased stress on the spine and pelvis, forward shifting of the female’s center of gravity, and increased joint laxity of the pelvis [30]. The prevalence of low back pain in the pregnant population is roughly 50-75% [30]. The type of WMSDs for female physicians during pregnancy was not specified. However, there may be a higher incidence of lower back pain versus upper extremity pain during pregnancy [21].

Ergonomic Training and Intraoperative Breaks

Several studies expressed the need for early-career ergonomic training throughout many procedural specialties [1,8,14,16,21,25,27,28,38]. When inquired on ergonomic knowledge and training, 62-76% of physicians had not received formal training or recommendations on proper ergonomics to reduce the development of WMSDs [14,21,38]. Additionally, studies discovered that many surgeons do not have access to ergonomic equipment or proper room design in the operating room [15,28,38]. Giagio et al. conducted a randomized controlled clinical trial among 141 surgeons across multiple specialties including urology and orthopedic, vascular, general surgery who were randomized into a preventive program (n = 65) or non-preventive program (n = 76) as the control group [39]. The preventive program consisted of six months of physical therapy training on ergonomic principles and exercises. Ergonomic implementation in the operating room consisted of optimal monitor and table positioning and the use of lead aprons, sitting stools, and floor mats. At the end of the trial, there was a statistical improvement in surgeon quality of life at three and six months (*P* < 0.04), with a significant reduction of lower back pain at six months (*P* < 0.01) [39]. This study highlights the importance of ergonomic education programs in reducing WMSDs and improving quality of life. Incorporating ergonomic courses into residency and fellowship training can help decrease the risks of developing WMSDs. Using specific ergonomic positions to alleviate strain based on the locations of pain experienced by physicians could help target areas of increased prevalence, such as the lower back for men and the upper extremity for women. 

In addition to physical and room design recommendations, incorporating intraoperative breaks can help to reduce WMSDs. Fifty-one to eighty-three percent of physicians took breaks during endoscopic procedures, and their relief methods were adjusting table height, stretching, exercising, and rest [15,23,24]. In Pawa et al., those who took breaks (15-30 minutes) or micro-breaks (30 seconds to two minutes of meaningful movements) during procedures had a significantly lower likelihood of developing ERI, with no significant difference between taking a break or micro-break (*P* < 0.002) [21]. Park et al. analyzed the utility of incorporating micro-breaks during surgical operations [40]. The study comprised 66 physicians in the specialties of obstetrics-gynecology, urology, and general, colorectal, and orthopedic surgery. The surgeons operated without a micro-break, then performed another case with a micro-break. This study’s definition of a micro-break was a standardized 90-second-to-two-minute session of targeted stretching every 20-40 minutes during an operation. The utilization of intraoperative micro-breaks was found to significantly improve pain scores of the neck, shoulders, and hands. Eighty-seven percent of the physicians planned to incorporate micro-breaks into their practice [40]. With the increased risk of upper extremity pain in female physicians, incorporating focused stretching of the shoulders and hands during procedures could help prevent the development of work-related injury.

WMSD Prevention in IR

Several ergonomic recommendations provided for interventional radiologists were either regarding physical adjustment or room design. The physical recommendations include good core strength, upright posture, intermittently resting a foot during lengthy procedures, using a lumbar support belt, and wearing appropriately fitted lead [1,4,5]. Room design suggestions consist of having the monitor set to 15 degrees below the vertical gaze, having the table at elbow level with arms held at 90 degrees, positioning the C-arm between the physician and the monitor, use of floor mats, and utilizing freestanding or suspended radiation shields [1,4,5]. While these ergonomic recommendations are helpful, it is imperative that training on these techniques should be incorporated into the IR curriculum, especially with the more recent establishment of integrated IR residency programs [1]. A better understanding of the types of WMSDs experienced by female physicians can help adjust the approach to ergonomic training for women.

Limitations

This review has several limitations, with the most notable being the variability in ergonomic questionnaires measured in each study. A standardized assessment regarding the physician-reported WMSDs would have generated greater significance and more accurate comparisons among the studies. Additionally, voluntary response bias is a limitation when evaluating questionnaire data, which may provide evidence that deviates from the general population of physicians experiencing WMSDs. The low rate of responses and analysis of female physicians among these studies hindered our investigation of locations of WMSDs developed in women. 

## Conclusions

In summary, there was poor female representation, averaging 25.7% of respondents among these 11 studies. Several characteristics were that women were generally shorter, wore smaller glove sizes, and were younger than their male colleagues. Seventy-two percent of female proceduralists reported WMSDs versus 46.6% of their male colleagues. Furthermore, women may experience more upper extremity pain than lumbar pain, which men commonly reported. Potential contributing factors to WMSDs are the size and design of procedural tools and the possible predisposition of female physicians to experience upper extremity WMSDs while performing the same operations as men. As more women enter medicine and pursue careers in procedural fields like interventional radiology, it is essential to address these discrepancies and develop ergonomically sound solutions for women.

Future directions

To better understand the differences in WMSDs among female interventionists, a survey of interventional radiologists that includes women in IR is essential. Through a comprehensive survey, we could identify more information on female interventionists’ prevalence, location, severity, and causes of WMSDs. Conducting a trial that implements a preventive program in IR based on the current ergonomic recommendations would provide insight into practical ways of reducing WMSDs for both women and men. With the innovative nature of IR, future medical devices must consider ergonomics, anthropometry, and physician usability of various sizes in their design. With the rise in integrated interventional radiology residency programs, it is essential for ergonomic training to begin in early career development. Incorporating these techniques at the beginning of IR training could significantly diminish the occurrence of WMSDs in the future, improving quality of life, rates of burnout, and overall physician satisfaction.
